# An Engineered N-Cadherin Substrate for Differentiation, Survival, and Selection of Pluripotent Stem Cell-Derived Neural Progenitors

**DOI:** 10.1371/journal.pone.0135170

**Published:** 2015-08-05

**Authors:** Amranul Haque, Nihad Adnan, Ali Motazedian, Farhima Akter, Sharif Hossain, Koichi Kutsuzawa, Kakon Nag, Eiry Kobatake, Toshihiro Akaike

**Affiliations:** 1 Department of Biomolecular Engineering, Tokyo Institute of Technology, Yokohama, Japan; 2 Department of Biological Information, Tokyo Institute of Technology, Yokohama, Japan; University of Newcastle upon Tyne, UNITED KINGDOM

## Abstract

For stem cell-based treatment of neurodegenerative diseases a better understanding of key developmental signaling pathways and robust techniques for producing neurons with highest homogeneity are required. In this study, we demonstrate a method using N-cadherin-based biomimetic substrate to promote the differentiation of mouse embryonic stem cell (ESC)- and induced pluripotent stem cell (iPSC)-derived neural progenitor cells (NPCs) without exogenous neuro-inductive signals. We showed that substrate-dependent activation of N-cadherin reduces Rho/ROCK activation and β-catenin expression, leading to the stimulation of neurite outgrowth and conversion into cells expressing neural/glial markers. Besides, plating dissociated cells on N-cadherin substrate can significantly increase the differentiation yield via suppression of dissociation-induced Rho/ROCK-mediated apoptosis. Because undifferentiated ESCs and iPSCs have low affinity to N-cadherin, plating dissociated cells on N-cadherin-coated substrate increase the homogeneity of differentiation by purging ESCs and iPSCs (~30%) from a mixture of undifferentiated cells with NPCs. Using this label-free cell selection approach we enriched differentiated NPCs plated as monolayer without ROCK inhibitor. Therefore, N-cadherin biomimetic substrate provide a powerful tool for basic study of cell—material interaction in a spatially defined and substrate-dependent manner. Collectively, our approach is efficient, robust and cost effective to produce large quantities of differentiated cells with highest homogeneity and applicable to use with other types of cells.

## Introduction

Unlike peripheral nervous system (PNS), neurons in the central nervous system (CNS) do not spontaneously regenerate injured axons because of extrinsic inhibitory factors and intrinsically lower growth capacity [1,2]. Conditioning neurons by neural extracellular matrix (ECM) components and cell adhesion molecules (CAMs) are thought to play an important role in increasing the intrinsic growth capacity of neurons and neurites both *in vitro* and *in vivo* [3,4]. Furthermore, during embryonic development, ECMs and CAMs play a major role in the formation and expansion of the neural crest and neural tube that finally results in PNS and CNS, respectively [5–7].

The extracellular part of neural CAM (N-cadherin) typically mediates calcium-dependent homophilic interaction and modulates several signaling pathways including Akt, Wnt/β-catenin, fibroblast growth factor (FGF)-2, and Rho GTPases [8–12]. During neurogenesis, N-cadherin plays important role in axon outgrowth [13], dendritic branching [14], synaptogenesis [15], and synaptic plasticity [16–18]. In a number of studies, molecular tethering of CAMs and growth factors (GFs) has been proposed to understand key developmental signaling pathways by increasing protein stability, promoting persistent signaling, and reducing complexities associated with *in vivo* microenvironment [19–22]. Despite the emphasis given to biological surface modification in order to mimic pluripotent stem cell microenvironment, few studies have utilized these modified surface for controlling stem cell differentiation in a spatially defined and substrate-dependent manner.

This study began with an observation that, when cultured on surfaces pre-coated with recombinant mouse N-cadherin-Fc chimera (termed N-cad-Fc throughout this paper) in the absence of exogenous neuro-inductive signals, embryonic stem cell (ESC)- and induced pluripotent stem cell (iPSC)-derived neural progenitor cells (NPCs) showed remarkable enhancement in neurite formation compared to cells cultured under identical conditions on substrates commonly used for neuronal cell culture. To the best of our knowledge, such enhancement in neurite extension and neuronal conversion has not been observed previously for ESC- and iPSC-derived NPCs differentiated without exogenous GFs or inhibitors. The molecular mechanism underlying such effects is associated with reduced Rho/ROCK activation and β-catenin expression.

Additionally, we presumed that plating dissociated cells versus neurospheres (cluster of NPCs) would also significantly increase the homogeneity of differentiated neural cells, as shown previously by Barde and coworkers [23,24]. However, most of the conventionally used extracellular matrices do not have selectivity to particular cell types. Also, many cell types including ESCs [25], ESC-derived NPCs [26], intestinal stem cells [27], and keratinocytes [28] are susceptible to dissociation-induced RhoA/ROCK-mediated apoptosis. These are two major obstacles associated with the derivation of differentiated cells in high yield and purity. Even though, it has been reported that a selective ROCK inhibitor is capable of increasing survival and cloning efficiency of dissociated single cells *in vitro* [25], the chemicals or inhibitors necessary for stem cell culture and differentiation require strict monitoring of all critical aspects classically associated with embryotoxicity and cytotoxicity [29]. To circumvent these obstacles, first, we dissociated neurospheres into single cells by the traditional enzymatic methods and secondly, we plated dissociated cells on a surface coated with N-cad-Fc. We found that N-cadherin-mediated suppression of Rho/ROCK signaling markedly diminishes dissociation-induced apoptosis, while cadherin-dependent homophilic interaction allowed us to isolate NPCs from a heterogeneous mixture of various cell types.

## Materials and Methods

### Ethics Statement

All animal experiments were performed with the approval of the Animal Ethics Committees of Tokyo Institute of Technology and in accordance with the Ethical Guidelines for Animal Experimentation of Tokyo Institute of Technology. Four-weeks-old BALB/c mouse brain was used as control.

### Cell culture

The culture parameters for feeder-dependent mouse ESC line ST1 [30] and Nanog-GFP expressing iPSC line (APS0001 iPS-MEF-Ng-20D-17, RIKEN BioResource Center) have been explained in details elsewhere [30,31]. In brief, ST1 and iPSCs cell lines were cultured on irradiated mouse embryonic fibroblast (CF-1 MEF, GlobalStem), grown on gelatin-coated dish, in a humidified atmosphere of 5% CO_2_ at 37°C. The cell culture medium for ST1 cells was composed of Dulbecco’s Modified Eagle’s Medium (DMEM; Sigma-Aldrich) supplemented with 20% (v/v) fetal bovine serum (FBS; Biochrom AB), 1mM L-glutamine, 1% nonessential amino acids (NEAA; Gibco, Life Technologies), 1 mM sodium pyruvate (nacalaitesque), 0.1 mM 2-mercaptoethanol (Sigma Chemical), 1000 units/ml recombinant leukemia inhibitory factor (LIF; Chemicon, Millipore), 50 μg/ml penicillin, and 50 μg/ml streptomycin (nacalaitesque). iPSCs were maintained in the same self-renewal media used for ST1, except that iPSC medium was supplemented with 15% FBS. ESCs and iPSCs were passaged in every third day with daily media change.

### Cell culture dish coating

Control plates: tissue culture plates (Iwaki) were coated for 30 min at 37°C with sterile solution of 0.1% (w/v) gelatin type A (Sigma Chemical) and 100 μg/ml poly-L-lysine (PLL; Sigma-Aldrich). PLL coated surface was washed with sterile water and allowed to dry. Glass-bottom 35 mm dishes (Matsunami) were used for confocal imaging. Cadherin coating: expression, purification and immobilization of E-cad-Fc and N-cad-Fc on non-treated tissue culture polystyrene dishes have been explained in details elsewhere [30,32]. Briefly, to prepare cadherin-Fc-coated surface non-treated polystyrene dishes (Iwaki) were treated with 10 μg/ml of purified cadherin-Fc and incubated for 1 h at 37°C. The spent solutions were discarded and surfaces were rinsed once with PBS. Prepared plates were used immediately.

### Induction of differentiation

Basal differentiation medium (BDM) for ESCs and iPSCs contained Glasgow Minimum Essential Medium (GMEM, Sigma-Aldrich) supplemented with 10% (v/v) knockout serum replacement (KSR; Gibco), 1 mM sodium pyruvate, 1 mM L-glutamine, 1% NEAA, 0.1 mM 2-mercaptoethanol, 50 μg/ml penicillin, and 50 μg/ml streptomycin. The differentiation induction and maturation factors that were used in this study were as follows: CKI-7 (Sigma-Aldrich), SB431542 (Wako) and basic fibroblast growth factor (bFGF;Promega). Before induction of differentiation, ESCs and iPSCs were cultured on E-cad-Fc-coated plates for two or three passages to remove feeder cells. For the induction of ESCs and iPSCs into NPCs, neurospheres were generated by suspension culture. For suspension culture-based differentiation, ESCs/iPSCs cultured on E-cad-Fc were treated with Accutase (Innovative Cell Technologies) for 3 min at 37°C. Dissociated cells were resuspended in BDM at a density of 5x10^4^ cells/35 mm dish. The day on which cells were seeded to differentiate was defined as day 0. In the differentiation protocol, BDM containing 5 μM of CKI-7 and 5 μM of SB431542 were used for the first 3 days of differentiation. On day 3, medium was changed to fresh differentiation medium with half the initial concentration of CKI-7 and SB431542 (abbreviated CK/SB throughout this paper). The neurospheres were cultured for 2 more days. Medium was changed every two days and bFGF (20 ng/ml) was used between 10 and 20 days of differentiation. In suspension culture technique, neurospheres were also generated spontaneously in BDM. This GF/inhibitor-free differentiation protocol was used to further elucidate the neuro-inductive properties of substrate bound N-cadherin.

### Neurite outgrowth assay

For qualitative analysis, axons outgrowing from the neurospheres were confirmed by β-III tubulin (Tuj) expression. For neurite outgrowth assay, neurite outgrowth surface (Sn) and neurosphere surface (Se) were measured using the Metamorph software (Universal Imaging Corporation) and a neurite extension index (Sn/Se) was calculated, as initially described by Matsunaga et al [33]. Time-lapse imaging was performed to show a phase-contrast movie of differentiated cells 24 h after plating on N-cadherin substrate at 135 frames, one frame per 10 min, with parameter set as each pixel equivalent to 1 μm.

### Inhibition, activation, and viability assays

For inhibition and activation assay, the neurospheres were incubated for 1 h in the presence of FGFR signaling inhibitor, PD173074 (75 nM; Sigma-Aldrich); ROCK inhibitor, Y-27632 (10 μg/ml; Sigma-Aldrich), and RhoA activator I, CN01 (1 U/ml; calpeptin; Cytoskeleton, Inc.). The intact or dissociated neurospheres were then transferred on ECM molecules and cultured for additional 2 days in the presence of these inhibitors and activator. Control experiments without inhibitors and activator were carried out in parallel. For viability assay, the dissociated neurospheres on day 5 were plated at a density of 50,000 cells per cm^2^ on ECM-coated plates in the presence and absence of Y-27632 and left to adhere for 4 hours in a cell incubator. Subsequently, the plates were washed three times to remove the non-adherent cells followed by enzymatic dissociation of adherent cells. The dissociated cells were stained with 0.4% Trypan Blue (Bio-Rad) to get initial seeding density for normalization. The cells cultured for 48 h were dissociated by following the same approach mentioned above and used for survival assay using TC20 Automated Cell Counter (Bio-Rad). All the experiments were performed in triplicates.

### Immunocytochemistry

Cells were fixed with Mildform 20N (8% formaldehyde; Wako) for 30 min and permeabilized with 0.2% Triton X-100 (nacalaitesque) for 5 min. Fixed cells were blocked with Blocking One solution (nacalaitesque) for 1h. The primary antibodies used here were: rabbit anti-human Nestin (1:200; N-1602; IBL Ltd.), mouse anti-neuron specific βIII-tubulin antibody (1:500; TuJ-1; R&D Systems), mouse monoclonal anti-MAP2 antibody (1:500; Sigma-Aldrich) and anti-mouse β-catenin (1:1000; BD Bioscience, USA). The samples were washed for three times, and incubated with goat anti-mouse IgG F(ab’)2-TRITC (1: 100; Santa Cruz) and anti-rabbit IgG F(ab’)2 Alexa Fluor 555 conjugate (1: 1000; Cell Signaling Technology) at room temperature for 1 h. After three times of washing, the samples were counterstained with 4’-6-diamidino-2-phenylindol (DAPI, Invitrogen), and examined by confocal inverted microscope (Nikon Eclipse *Ti*, Japan). Fluorescent images of approximately 400 cells in at least 3 different areas were analyzed for Tuj-positive cells. DAPI was used to quantify the total number of cells.

### Western-blot analysis

The total cellular proteins were extracted with a lysis buffer (20 mMTris-HCl, pH 8.0, 50 mMNaCl, 1% Nonidet P40 (Sigma), 1% Triton X100), 1% (v/v) phenylmethanesulfonyl fluoride (PMSF, Sigma), and 1% (v/v) protease inhibitor cocktail (nacalaitesque) (PI; pH 7.4). The cell lysates were centrifuged at 15,000g for 15 min at 4°C to achieve protein sample solutions. Proteins with sample buffer were separated by sodium dodecyl sulfate polyacrylamide gel electrophoresis (SDS-PAGE) on polyacrylamide gels (7.5% and 10%) and electrophoretically transferred onto a polyvinylidenedifluoride (PVDF; Immobilon- P, Millipore) membrane. The membranes were incubated with primary antibodies of mouse anti-β-actin (1: 2000; Sigma-Aldrich), anti-pAkt (1: 1000; Cell Signaling), total-Akt (Cell Signaling) and β-catenin (1: 2000; BD Bioscience) for 2 h at room temperature. The incubation with horseradish peroxidase (HRP)-conjugated secondary antibody (1:10,000 dilution; Jackson ImmunoResearch Laboratories) was performed for 2 h at room temperature. HRP activity was assayed with the Immobilon Western detection reagents (Millipore) according to the manufacturer’s instruction.

### Pull-Down Assay

Effector pull-down assay were performed using active RhoA binding Rhotekin-RBD protein GST beads (Cytoskeleton, USA) [34]. Cells were lysed using cell lysis buffer and the lysates were rotated for 1 hour at 4°C with 90 μg of either active RhoA binding Rhotekin-RBD protein GST beads (Cytoskeleton, USA). Following incubation, the beads were centrifuged and washed with washing buffer (25 mM Tris pH 7.5, 30 mM MgCl_2_, 40 mM NaCl) three times. Bound Rho proteins were eluted by incubation in Laemmli lysis buffer at 95°C for 7 min and subjected to SDS-PAGE and Western blot analysis.

### Real-time quantitative PCR

Total RNA from the cells were extracted using Trizol reagent (Ambion, Thermo Scientific). cDNA was synthesized using 0.2 μg of total RNA in 20 μl reaction mixture, containing oligo-dT primer using Moloney murine leukemia virus (M-MLV) reverse transcriptase (Invitrogen, Life Technologies), according to the manufacturer’s instructions. Real-time PCR was performed with SYBR Green (Applied Biosystems, Life Technologies) on Thermal Cycler Dice Real Time System Single and the results were analyzed by its software. All reactions were done in duplicates. Mouse brain was used for normalizing the expression of neuronal markers, while undifferentiated ESCs- or iPSCs were used to normalize Nanog (pluripotency marker) expression. Primers used are listed in S1 Table.

### Flow cytometry

The iPSC-derived neural progenitors were harvested with Accutase and analyzed. The dissociated 1x10^6^ cells/ml were ashed with cold PBS. The Nanog-GFP expression was then analyzed using flow cytometer (Guava Technologies, Millipore).

### Statistical analysis

Data are presented as the mean±standard deviations (SD). Statistical analyses were performed with Student’s t-test for paired samples. A p-value < 0.05 was considered statistically significant.

## Results

To directly investigate the functional involvement of N-cadherin in the regulation of neurite growth and neural conversion from ESCs and iPSCs, we used N-cadherin extracellular domain-Fc fusion protein (N-cad-Fc). This fusion aimed at producing a constitutive dimeric form of the extracellular region of N-cadherin, which can be used in a controlled fashion to mimic N-cadherin dependent cell—cell contact. To achieve ESC/iPSC conversion to their terminal differentiation into neural and glial cells in an efficient and reproducible way, we generated neurospheres from ESCs and iPSCs in suspension in the presence and absence of neuro-inductive signals, CK/SB (S1 Fig). We followed previously reported neural differentiation protocols [35,36] with minor modifications by eliminating signals (serum and activin A), which induce multi-lineage differentiation [37]. The NPCs generated under serum-free condition were transferred on N-cadherin substrate and cultured additional days for maturation.

### Effects of N-cadherin substrate on neurite outgrowth

**Fig 1:**
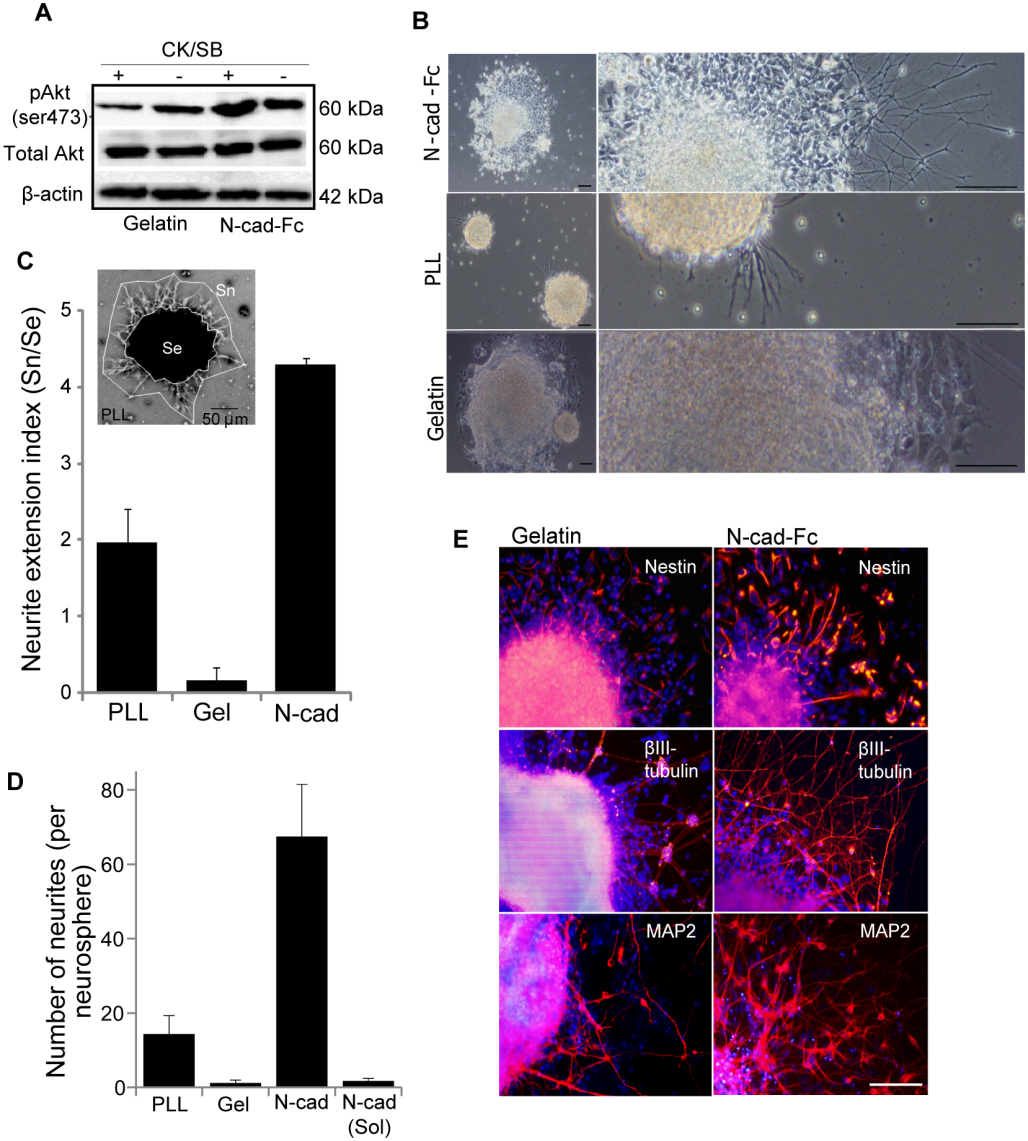
Enhancement of neurite outgrowth by substrate bound N-cadherin. Neurospheres generated in suspension for 5 days with and without CKI-7/SB-431542 were cultured on substrates pre-coated with N-cad-Fc, PLL, or gelatin for 48 hrs in the absence of growth factors. **(A)** N-cadherin substrate leads to upregulation of Ser473P (active) Akt in NPCs, as analyzed by western blot. **(B)** Cellular phenotype on different extracellular matrices was assessed by bright-field microscopy. **(C)** The degree of neurite outgrowth on the N-cad-Fc, gelatin, and PLL was quantified by neurite extension index, defined as the ratio of neurite outgrowth surface (Sn) and explant surface (Se). **(D)** The number of neurites per neurosphere was analyzed from bright-field images. Soluble N-cad-Fc was added in the growth medium of explants grown on gelatin substrate. **(E)** Immunofluorescence microscopy analysis of Nestin, βIII-tubulin, and MAP2 expression. DAPI shows total nuclei in the field of view. Data are mean ± SD, n = 3. Scale bar is equivalent to 50 μm unless otherwise mentioned. Abbreviation: PLL- poly-L-lysine; sol, soluble; Ncad, N-cad-Fc; Gel, gelatin.

Studies imply that Akt activation by N-cadherin induces neuronal differentiation during cortical development [8]. To test the functionality of immobilized N-cadherin ectodomain through Akt activation, NPCs generated in the presence and absence of CK/SB for five days were plated in tissue culture dishes coated with N-cad-Fc and gelatin. As expected, cells seeded on N-cadherin showed increase in Ser473P Akt activation than gelatin controls (Fig 1A). We then assessed the effect of matrix tethered N-cadherin on neurite outgrowth. ESC/iPSC-derived neurospheres plated on N-cadherin-coated surface showed dramatic enhancement in neurite outgrowth than controls (Fig 1B). While it took at least 32 h to see any visible outgrowth on gelatin- or PLL-coated surfaces, neurospheres on N-cadherin substrate formed neurites within 18 h of culture, suggesting a faster growth rate on N-cadherin (data not shown). We have also used time-lapse video microscopy to confirm the growth of neurites on N-cadherin substrate (S1 Movie). These observations are supported by previous reports that local stimulation with extrinsic N-cadherin is sufficient for the activation of earliest signals involve in the formation of neurites [38]. The influence of N-cadherin was further analyzed quantitatively by previously reported neurite outgrowth assay [33] using a neurite extension index, defined as the ratio of neurite outgrowth surface (Sn) and explants surface (Se). Fig 1C shows that neurospheres cultured on N-cadherin substrate for 48 h formed 2- and 40-times longer neurites than spheres on poly-L-lysine (PLL) and gelatin, respectively. The neurite extension index on N-cad-Fc, PLL and gelatin were 4.20±0.084, 1.96±0.45 and 0.16±0.17, respectively. The differences in extension index were more dramatic while cells were cultured for additional days on these substrates. Compared to PLL and gelatin, the number of neurites on N-cad-Fc was also 4- and 60-fold higher, respectively (Fig 1D). With regard to the role of soluble N-cadherin ligand in axonal growth, evidence suggested both a stimulatory and a trivial effect on primary neural cells [39,40]. In this study, we did not observe any significant increase in neurite outgrowth when soluble N-cad-Fc was added in the differentiation medium of neurospheres grown on gelatin substrate (Fig 1D).

In addition to bright-field images, outgrowth was also evaluated by staining with microtubule-associated protein (MAP)-2 in ESC- and iPSC-derived NPCs, which were stained positive for neuronal cytoskeletal antigen (class III β-tubulin isotype or βIII-tubulin) and nestin (Fig 1E). Different from gelatin, outgrowth on N-cadherin substrate was more robust and showed less bundles with spreaded curvilinear trajectories. Taken together with previous findings [38,41], these results support the notion that substrate bound N-cadherin is extremely potent in inducing neurite outgrowth than other CAMs (e.g., L1) and ECM proteins (e.g., gelatin, collagens, and laminin). Overall, these data demonstrate the profound effect of cadherin-based biomaterial substrate to accelerate the process of neurite growth from ESC- and iPSC-derived NPCs. Later in this study we will confirm the mechanism underlying enhancement of neural conversion on N-cadherin substrate.

### Neuronal differentiation on N-cad-Fc without exogenous growth factors and inhibitors

**Fig 2:**
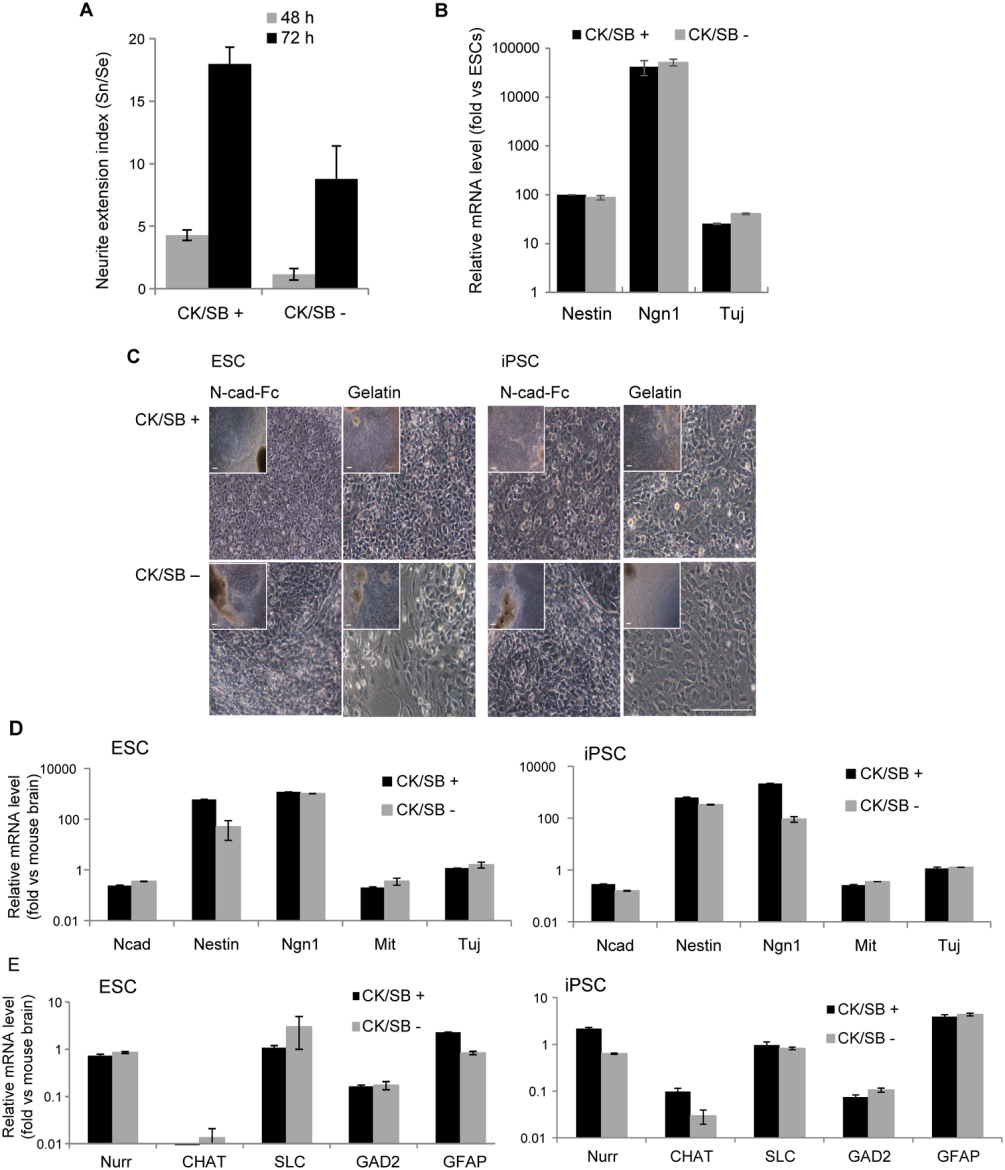
Neuronal differentiation on N-cadherin substrate without exogenous growth factors or inhibitors. **(A-B)** ESCs cultured in suspension for 5 days with and without CKI-7 and SB-431542 (CK/SB) was further induced on N-cadherin substrate for 48 h in the absence of exogenous growth factors. The degree of neural conversion was analyzed by **(A)** neurite extension index and **(B)** qRT-PCR for relative mRNA expression of nestin, neurogenin 1 (Ngn1), and βIII-tubulin (Tuj). **(C)** Cellular phenotype of differentiated cells on N-cad-Fc and gelatin substrates at 20 days of differentiation was assessed by bright-field images. Scale bar: 200 μm. **(D)** Characterization of neural cells at 20 days of differentiation by the expression of neuronal progenitor cell markers, including N-cad, Nestin, Ngn1, Mitf and Tuj. **(E)** The relative mRNA expression level of neurotransmitter and subtype-specific genes (Nurr, CHAT, SLC, GAD2 and GFAP) in ESC- and iPSC-derived neuronal cells differentiated for 20 days on N-cadherin substrate. Abbreviation: ESCs, embryonic stem cells; iPSCs, induced pluripotent stem cells; Ngn1- Neurogenin 1; Mitf- Microphthalmia-associated transcription factor; N-cad, N-cadherin; Nurr- nuclear receptor-related factor; CHAT- choline acetyltransferase; SLC- solute carrier; GAD- glutamate decarboxylase; GFAP- glial fibrillary acidic protein.

Default differentiation of mouse ESC to neuroectoderm occurs when culturing cells in serum-free medium and in the absence of any exogenous GFs [37,42]. Previous reports have also shown a vital role of endogenous signals in inducing mesoderm and endoderm lineages when differentiated without exogenous GFs [43,44]. Therefore, we sought to investigate if the effects of N-cadherin substrate on neurite growth and neuronal conversion could be replicated without exogenously added soluble GFs and inhibitors. Compared to CK/SB-free differentiation, a significant enhancement in neurite outgrowth was observed when differentiations were performed under the guidance of CK/SB (Fig 2A). Surprisingly, the expression level of well described markers for NPCs including nestin, neurogenin 1 (Ngn1), and βIII-tubulin (Tuj) was similar at day 7 in both conditions, as analyzed by qRT-PCR (Fig 2B).

To gain further molecular insight into these observations, adherent differentiation paradigm was assessed on day 15 (i.e., 20 days of differentiation including suspension culture) for subtype specific genes known to be responsible for different aspects of neuronal differentiation and lineage commitment. First, we noticed that cells on N-cadherin and gelatin substrate proliferate rapidly at or near the periphery of the sphere and grow as a monolayer leaving small spheres at the center (Fig 2C). As expected, differentiated cells on N-cadherin were phenotypically more homogeneous than cells on gelatin. Compared to CK/SB-free differentiation, bright-field images show slightly better homogeneity in differentiated cells induced with CK/SB. To confirm these observations, qRT-PCR was performed to analyze neuronal progenitor genes including *N-cad*, *Nestin*, *Ngn1*, *Mitf* and *Tuj* (Fig 2D). Similar to Fig 2B, no significant difference was observed between differentiated cells cultured with and without CK/SB. Of note, the expression of neuronal markers on N-cadherin substrate was higher than differentiated cells on gelatin substrate (S2 Fig).

We also characterized the functional phenotype of neuronal cells on substrate bound N-cadherin based on the expression of neurotransmitter genes including *Nurr* (dopaminergic), *CHAT* (cholinergic), *SLC* (glutamatergic), and *GAD2* (GABAergic). GFAP was used as glial cell marker. Consistent with previous observations, similar levels of transcripts for neurotransmitter genes were found at day 20 in ESC/iPSC progeny differentiated with or without CK/SB (Fig 2E). Of note, the expression of *CHAT* was lowest among other neurotransmitter genes, though recent reports indicate that N-cadherin induces partial differentiation of cholinergic presynaptic terminals in heterologous cultures of brainstem neurons and CHO cells [45]. Cells at day 20 also expressed GFAP, as gauged by qRT-PCR.

**Fig 3:**
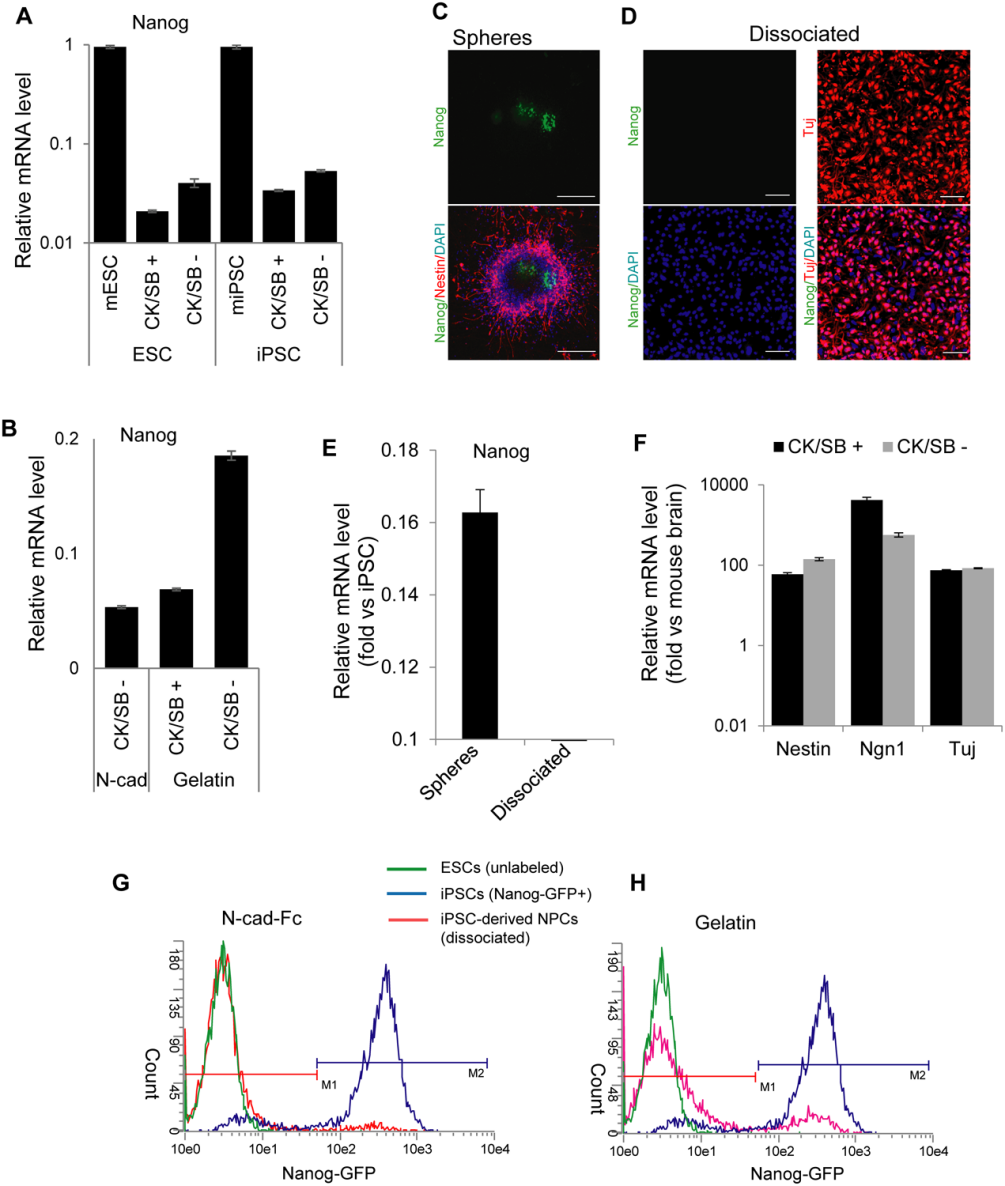
N-cadherin homophilic interaction enrich neuronal cells by eliminating undifferentiated ESCs/iPSCs. **(A)** The presence of Nanog expression in ESC- and iPSC-derived differentiated cells at 20 days of differentiation, as analyzed by qRT-PCR. Nanog expression was detectable regardless of the presence and absence of CK/SB. **(B)** qRT-PCR analysis for the expression of Nanog in differentiated cells cultured on N-cad-Fc and gelatin at day 20. **(C)** Nanog-GFP expressing iPSCs-derived neurospheres generated in suspension with CK/SB were seeded on N-cad-Fc shows the presence of Nanog (green) and Nestin (red) at 7 days of differentiation. **(D)** iPSC-derived neurosperes grown in suspension with CK/SB were enzymatically dissociated and plated on N-cadherin substrate for 48 h. Fluorescence images shows the absence of Nanog-GFP and the presence of Tuj (red). **(E)** Different from neurosphere-based differentiation, qRT-PCR analysis shows the absence of Nanog in dissociated cells cultured on N-cadherin for 48 h (total 7 days of differentiation). **(F)** mRNA expression analysis of Nestin, Ngn1 and Tuj expression in iPSC-derived progeny derived with or without CK/SB. Dissociated cells on N-cadherin substrate were harvested for analysis on 7 days of differentiation. **(G, H)** FACS analysis of Nanog-GFP expressing iPSCs. Neurospheres on day 5 were dissociated and cultured on N-cad-Fc and gelatin substrate for 48 h before analysis. DAPI shows total nuclei in the field of view. Scale bar: 50 μm.

At this stage rather than separating subtype specific neuronal and glial cells, which act synergistically to maintain physiological function [46], we put the focused on the isolation of neural/glial cells from heterogeneous cell populations by analyzing the markers for pluripotency (Nanog), mesoderm (Brachyury, Bra), and endoderm (albumin, Alb) lineages. Compared to mouse brain as negative control, the expression of markers for mesoderm and endoderm lineages was almost null in differentiated cells cultured with and without CK/SB (S3 Fig). We infer from this and other studies that the removal of serum that contains poorly defined endoderm- and mesoderm-inducing factors together with early differentiation induction under adhesion-free condition preferentially induced neuronal lineages [47]. However, we cannot oversee the cumulative data suggesting the obvious presence of residual undifferentiated cells associated with neurosphere- or embryoid body-based differentiation protocol [42,48]. Not surprisingly, the expression of Nanog was detectable in all conditions regardless of the presence and absence of CK/SB (Fig 3A). Compared to N-cadherin substrate, Nanog expression was higher in cells on gelatin substrate. While CK/SB was not added into the differentiation media the expression of Nanog was increased by a factor of 2 and 30 for N-cadherin and gelatin substrates, respectively (Fig 3A and 3B). These data further confirm the neuro-inductive effect of substrate bound N-cadherin. We also checked the presence of Nanog-GFP expressing cells at day 7 in order to localize remaining undifferentiated cells. Interestingly, a cluster of cells expressing Nanog-GFP was observed at the innermost layer of the spheres, which was clearly distinguishable by their physical separation from nestin-expressing cells (Fig 3C). This data is not surprising considering the notion that some pluripotent cells escape differentiation and remain undifferentiated over months under neural differentiation condition [48]. Regardless, we found that the neural differentiation on gelatin was more heterogeneous than N-cad-Fc substrate and the presence of remaining undifferentiated cells was inevitable, irrespective of the addition of exogenous soluble neuro-inductive signals.

### N-cadherin-dependent homophilic interaction to eliminate remaining undifferentiated cells

Previously we reported an E-cadherin-based “matrix-dependent cell-sorting” approach to enrich endoderm-derived hepatic progenitors [22]. Here, we wanted to see if N-cadherin substrate can replicate similar sorting effect in order to enrich neuronal progenitors by eliminating undifferentiated cells. Of note, unlike neuronal cells, naïve ESCs and iPSCs do not express or poorly express cell surface N-cadherin [30]. In order to achieve highest homogeneity, iPSC-derived spheres grown in suspension without CK/SB were enzymatically dissociated at day 5 and cultured for 48 h on gelatin and N-cadherin substrates (S4 Fig). Immunostaining images show the absence of markers for pluripotency (Nanog), mesoderm (Brachyury), and endoderm (α-fetoprotein or AFP) lineages in differentiated cells, which were stained positive for Tuj (Fig 3D and S5 Fig). Likewise, qPCR analysis confirmed the absence of Nanog and presence of NPC markers in singly dissociated cells plated on N-cadherin substrate for 48 h (Fig 3E and 3F). It is noteworthy that more than 97% of dissociated cells on N-cadherin were Nanog-GFP negative (Fig 3G) and Tuj positive (Fig 3D), while 30% cells on gelatin expressed Nanog (Fig 3H), as determined by flow cytometry and immunofluorescence assay.

Besides the lack of selectivity, a massive cell death was observed while dissociated cells were plated onto the control substrates. We observed >50% cell death within 24 h of culture on gelatin, whereas the viability was increase to ~82% with ROCK-inhibitor, Y-27632. Strikingly, the viability of dissociated cells on N-cadherin cultured without Y-27632 was similar to those cells plated on gelatin with Y-27632 and increased to 95% in the presence of Y-27632 (S6 Fig). In the next section we will reveal the mechanism underlying the enhancement of neural conversion and suppression of dissociation induced apoptosis on N-cadherin substrate.

### Suppression of Rho/ROCK and β-catenin signaling pathways by N-cadherin substrate

**Fig 4:**
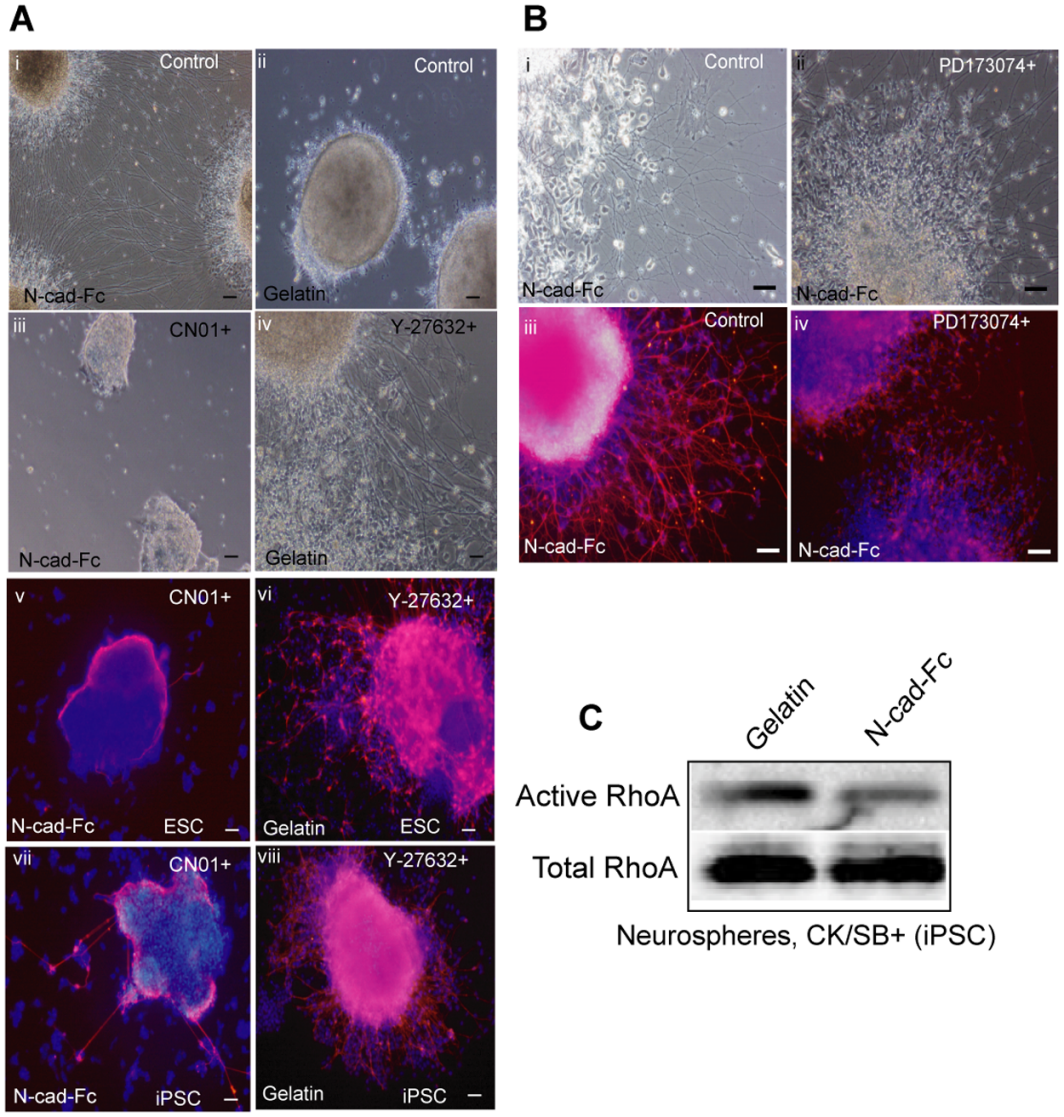
N-cadherin substrate reduces Rho/ROCK activation in ESC-/iPSC-derived neurospheres. ESCs and iPSCs were induced with CK/SB for 5 days and then plated on surface pre-coated with gelatin and N-cad-Fc for 48 h with and without ROCK inhibitor (Y-27632), RhoA activator (CN01) or FGF2-receptor antagonist (PD173074). **(A)** Bright-field images shows the enhancement of neurite extension on gelatin when cultured with Y-27632. Complete inhibition of neurite outgrowth was observed on N-cadherin substrate when cells were treated with CN01. The presence of neurite outgrowth was confirmed by staining with βIII-tubulin staining (red). **(B)** The degree of neurite extension on N-cadherin substrate was similar, regardless of the presence and absence of PD173074, as gauged by immunostianing of βIII-tubulin (red). **(C)** Pull-down assay of active RhoA protein in differentiated iPSCs. DAPI shows total nuclei in the field of view. Scale bar: 50 μm.

An extensive body of research has demonstrated that FGFR2, β-catenin and RhoGTPase signaling pathways controlled by N-cadherin can stimulate axon growth in primary neural and glial cells [8,9,11,12,40]. To test if Rho/ROCK signaling is involved in stimulating neurite outgrowth, we cultured neurospheres for 48 h in the presence of RhoA activator (CN01) on N-cad-Fc and ROCK inhibitor (Y-27632) on gelatin. The neurite outgrowth was markedly increased on gelatin in the presence of Y-27632 and decreased on N-cad-Fc with CN01 (Fig 4Ai–4Aiv). Besides bright-field images, the neurite outgrowth was confirmed by Tuj staining (Fig 4Av–4Aviii). Treatment with PD173074, a specific antagonist of FGF2R, did not affect the formation and extension of neurites (Fig 4B). To provide the direct evidence on the involvement of Rho proteins in stimulating neurite outgrowth on N-cadherin substrate, the relative cellular concentrations of activated RhoA protein was determined using effector pull-down assay exploiting the RhoA binding domain Rhotekin. The activation of RhoA on N-cad-Fc was lower than cells cultured on gelatin-coated surface (Fig 4C).

**Fig 5:**
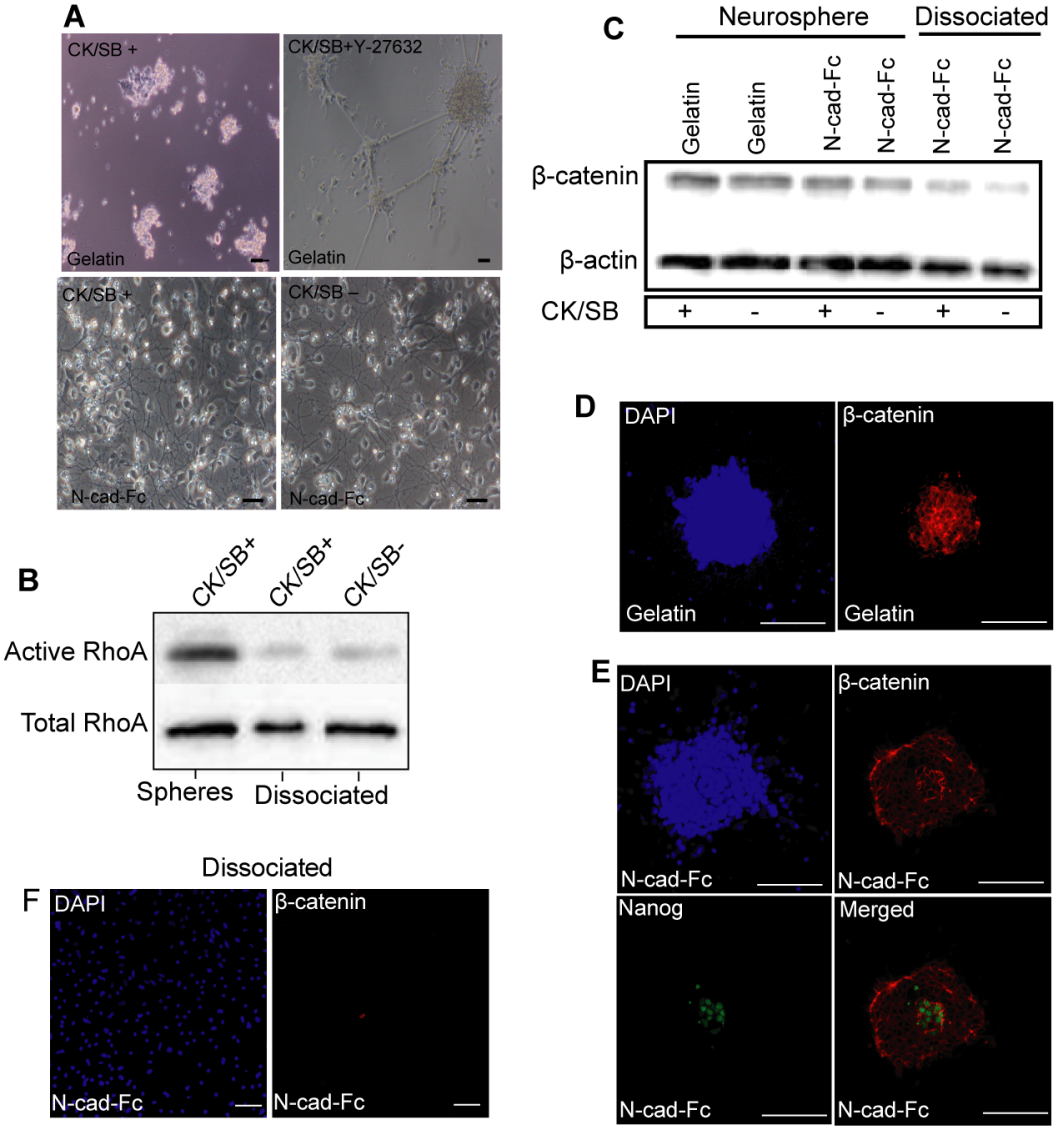
Suppression of Rho/ROCK and β-catenin signaling pathways by N-cadherin substrate in singly dissociated cells. iPSC-derived neurosperes grown in suspension with and without CK/SB were enzymatically dissociated at day 5 and cultured on N-cadherin and gelatin substrate for 48 h. **(A)** In contrast to N-cadherin substrate, cells on gelatin failed to form neurites in the absence of Y-27632, as analyzed by bright-field images. Cellular phenotype on N-cadherin substrate was similar irrespective of the presence and absence CK/SB. **(B)** Compered to spheres seeded on N-cadherin substrate, pull-down assay shows down-regulation of active RhoA in dissociated cells. **(C)** β-catenin expression was analyzed by western-blotting. Immunostaining images for the expression of β-catenin (red) in shepres plated on **(D)** gelatin, and (**E)** N-cad-Fc substrate. β-catenin (red) expression is higher in Nanog expressing cells (green). **(F)** β-catenin expression was undetectable in dissociated cells plated on surfaces pre-coated with N-cad-Fc. DAPI shows total nuclei in the field of view. Scale bar: 50 μm.

We also analyzed the effect of N-cadherin on Rho/ROCK signal activation in dissociated cells cultured on N-cadherin for 48 h without growth factors. As shown in Fig 5A, cells on gelatin were able to form neurites outgrowth in the presence of Y-27632. However, the differentiated cells on gelatin never achieved a true confluent culture nor do they form a homogeneous monolayer. In contrast, dissociated cells cultured on N-cadherin-coated surface attached well and formed a monolayer of cells with extended neurites in the absence of Y-27632. Similar effect was observed in cells induced with and without CK/SB. There were no differences with regard to cell shapes and neurite growth between cells growing on N-cadherin in the absence or presence of Y-27632 (data not shown). Pull-down assay also shows a significant down-regulation of RhoA activity in dissociated cells cultured on N-cadherin, irrespective of the addition of CK/SB in the early stages of differentiation (Fig 5B). These data suggest that N-cadherin stimulates neurite outgrowth by suppressing Rho/ROCK signaling.

It has been shown in cortical cells that N-cadherin can enhance neuronal differentiation and reduce neuronal precursor proliferation by down-regulating Wnt/β-catenin singling pathway [8]. To see whether ESC and iPSC-derived NPCs respond similarly to N-cadherin, we checked β-catenin expression level by western-blotting and immunostaning and confirmed that N-cadherin reduced the expression of β-catenin in NPCs (Fig 5C–5E). There was no noticeable differences found in β-catenin expression in NPCs generated with and without CK/SB (Fig 5C). Also, undifferentiated cells formed compact cellular aggregates and showed increased level of β-catenin in neurospheres (Fig 5E). Reasonably, the expression of β-catenin in dissociated single-cells on N-cadherin was nearly disappeared (Fig 5C and 5F), supporting previous finding on cardiac myocytes where Wnt signaling induces aggregation and adhesion by increased cadherin-β-catenin complex [49]. Fig 5E and 5F also suggest that plating dissociated cells on N-cadherin can selectively enrich NPCs by eliminating Nanog-expressing undifferentiated iPSCs. Taken together, substrate bound N-cadherin mediated down-regulation of RhoGTPase played vital role in enhancing neurite extension, neural conversion as well as neuroprotection against dissociation induced Rho/ROCK-mediated apoptosis.

## Discussion

**Fig 6:**
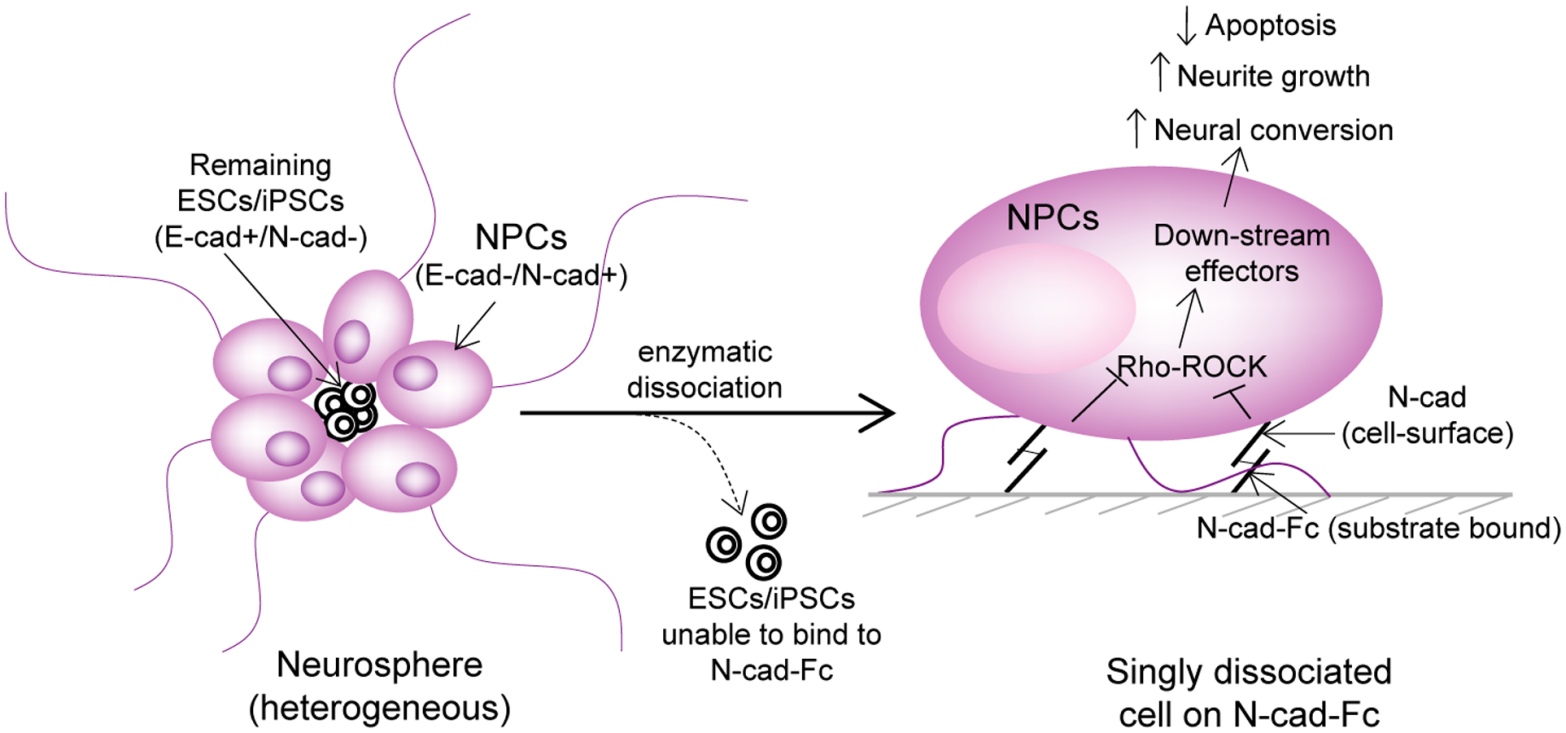
Proposed pathway connecting N-cadherin, Rho/ROCK, β-catenin signaling in neural differentiation. The neurospheres were heterogeneous; harboring undifferentiated ESC/iPSCs might be due to irregular distribution of soluble factors. To achieve better homogeneity, these spheres were dissociated and expanded on N-cadherin-coated surface. The down-regulation of β-catenin and Rho/ROCK pathways inhibited dissociation-induced apoptosis and enhanced neural conversion. N-cadherin mediated homophilic interaction is also implicated in the enrichment of neural population by eliminating undifferentiated ESC/iPSCs under serum/growth factor-free differentiation condition.

In contrast to previously reported neural differentiation protocols which typically rely on the addition of exogenous soluble factors, this study demonstrates an alternative method using N-cadherin-based biomimetic substrate to promote the differentiation of ESC- and iPSC-derived NPCs. We also identified the mechanism underlying the enhanced differentiation process on N-cadherin coated cell culture plate and showed that N-cadherin adhesion couples to RhoGTPase and β-catenin to accelerate the process of neurite growth and suppress dissociation induced apoptosis even in serum/GF-free condition. We showed that binding selectivity of cadherins can be useful to purge remaining ESCs/iPSCs from differentiated NPCs. Such a biomimetic system allows a precise control of spatial presentation of ligand molecules to the cells, which is not possible in natural contacts where different types of adhesion proteins can coexist with the lack of lineage selectivity. The experimental findings are summarized in Fig 6.

In general, the exogenous addition of GFs and small molecules improve the efficiency of ESC/iPSC differentiation into defined lineages [35,50]. It has recently been reported that endogenously secreted factors can control ESC self-renewal and differentiation. For example, cell secreted fibroblast growth factor 4 (FGF4) involve in generating neuroectodermal precursors [51]. Several other reports pointed to the vital role of cell secreted factors in inducing mesoderm and endoderm lineages under GF-free condition [43,44]. In the present study, homogeneous population of cells with neural phenotype were obtained by 10 days of differentiation in the absence of complex GFs, cytokines or inhibitors when grown on N-cadherin substrate. bFGF, by itself is not sufficient in controlling neural stem cell fate selection [51], was added in the NPC-maturation media between day 10 and 20 of differentiation. The intension behind using bFGF is simply to accelerate the differentiation process through enhancement of the activity of the GFs produced by the cells themselves [52]. The realization of the biomimetic substrate to control the differentiation process demonstrated here strengthens the growing body of literature indicating that biomaterials, much like the extracellular matrix, can direct stem cell fate [53,54].

In the present study, we also identified the mechanisms underlying the enhanced differentiation of NPCs on N-cadherin substrate. To mediate strong functional adhesion, cadherin must cluster, and the cluster is solely regulated by RhoGTPase and β-catenin. The exact role of β-catenin in cadherin-mediated axonal outgrowth remains to be clarified, as there is growing evidence suggesting both stimulatory and inhibitory influence of β-catenin on axonal growth and migration [9,55]. One reason for this discrepancy could be the lack of suitable assay condition. In the case single neuronal cells which were not involved in N-cadherin-mediated interaction showed inhibitory effect of β-catenin on neurite outgrowth, while cells in compact aggregates showed stimulatory influence. With N-cadherin coated surface we confirmed the inhibitory role of β-catenin on neurite outgrowth in dissociated NPCs (Fig 5C and 5F). Although the expression level of β-catenin was higher in cellular aggregates compared to dissociated NPCs, the overall expression level was low in both cases on N-cadherin compared to integrin-depended extracellular matrix molecules. The higher expression of β-catenin in cellular aggregates might be due to the presence of undifferentiated ESC which was confirmed by immunostaining of Nanog-expressing cells (Fig 5D). Regardless, we found the inhibitory role of N-cadherin in down-regulation of β-catenin signaling in ESC/iPSC-derived NPCs. Although these finding are related to primary neural cells, suppression of Rho/ROCK signaling increases neurite outgrowth. It is noteworthy that the neurospheres plated on N-cadherin substrate displayed reduced Rho/ROCK signal activation and the effect was most dramatic while singly dissociated cells plated on this substrate (Fig 5B). We assumed that a separation of the cells from each other forced a direct interaction of the cell surface N-cadherin with the matrix bound N-cadherin, thereby enhancing the effect of the substrate on the signaling molecules.

In this study, we did not generate sub-type specific mature neuronal or glial cells. It has already been reported that CK/SB can induce ESCs and iPSCs into functional retinal pigment epithelia cells [36,48]. Besides, the same group have reported the presence of residual undifferentiated cells in final differentiation outcome (retinal cells) even at day 40 of differentiation [48]. In order to develop safe stem cell-based treatments, the hurdle of tumorigenicity arising from undifferentiated cells must be addressed. Dissociation of neurospheres or multi-cellular colonies into single cells was a necessary step in the present method as these colonies generate heterogeneous populations by preserving Nanog-expressing undifferentiated cells (Fig 3C). However, dissociating neurospheres by the traditional enzymatic method resulted in cell survival rate lower than 20% on gelatin coated plates (S6 Fig). Together with dissociation-mediated apoptosis, lack of selectivity to particular cell type are major reasons of the failure of experiments in which singly dissociated cells are plated on ECM proteins (e.g., collagens, laminin, or gelatin) for better homogeneity. The exciting outcome of N-cadherin mediated down-regulation of Rho/ROCK signaling is the suppression of dissociation-induced apoptosis. We also explored the cell selection potential of N-cadherin substrate based on homophilic interaction. Because cadherins bind preferentially to similar cadherins (e.g., N-cadherin to N-cadherin), presentation of specific cadherins in an immobilized form will serve as a marker for isolation of homogeneous population of cells. Remarkably, with this approach we were able to generate pluripotent stem cell-free neuronal populations. Even though, undifferentiated ESC does not express or poorly express N-cadherin, the possible presence of N-cadherin expressing mesoderm populations cannot be overlooked on N-cadherin surface. Most interestingly, the expression of mesoderm- and endoderm-derived lineage markers was almost null. We suggest that the removal of serum containing endoderm- and mesoderm-inducing factors together with differentiation under adhesion-free condition preferentially induced ESCs- and iPSCs into neuronal cells on N-cadherin substrate.

Our findings on the effects of substrate-bound N-cadherin-dependent enhancement of neurite extension and neural conversion are consistent with reports published by our group [30] and Cherry et al. [56]. However, unlike these reports, this paper shows the influence of N-cadherin substrate on RhoGTPase and a transcriptional regulator, β-catenin in mouse ESC-derived NPCs. We also demonstrated a unique way to harness cadherin-based homophilic adhesion for cell selection and generation of better defined neuronal population in the absence exogenous soluble GFs and inhibitors.

## Conclusions

Our data suggest that N-cadherin exerts a neuritogenic effect in NPCs by enhancing neurite outgrowth and neural conversion via RhoGTPase and β-catenin signaling pathways. We also describe a label-free negative-selection approach to enrich NPCs simply by plating dissociated neurospheres on biomaterials substrata. Most importantly, the new method presented is not only a convenient and efficient way to enhance the viability and homogeneity of neuronal cells, but also demonstrates the successful strategy of using an appropriate biomaterial matrix to direct stem cell differentiation under completely defined and GF-free condition.

## Supporting Information

S1 Fig Schematic representation of the neuronal differentiation. (PDF)

S2 Fig Characterization of ESC-derived neuronal cells differentiated for 20 days on N-cadherin- and gelatin-coated substrate. (PDF)

S3 Fig Endoderm and mesoderm gene expression signature of differentiated ESCs. (PDF)

S4 Fig Schematic representation of the modified neuronal differentiation procedure from mouse ESCs/iPSCs in order to enhance the homogeneity of differentiation. (PDF)

S5 Fig Endoderm and mesoderm markers in differentiated cells upon dissociation. (PDF)

S6 Fig N-cadherin inhibited dissociation-induced-apoptosis in iPSCs. (PDF)

S1 Table Primer sequences used in qPCR. (PDF)

S1 Movie Time-lapse movie of neurite growth and migration from neurospheres on N-cadherin substrate. (AVI)
