# Supplementary material for: An Engineered N-Cadherin Substrate for Differentiation, Survival, and Selection of Pluripotent Stem Cell-Derived Neural Progenitors
Source: PLoS One. 2015 Aug 5;10(8):e0135170. doi: 10.1371/journal.pone.0135170 (PMC4526632; doi:10.1371/journal.pone.0135170)
Supplement: S1 Fig — (PDF) [file pone.0135170.s001.pdf]

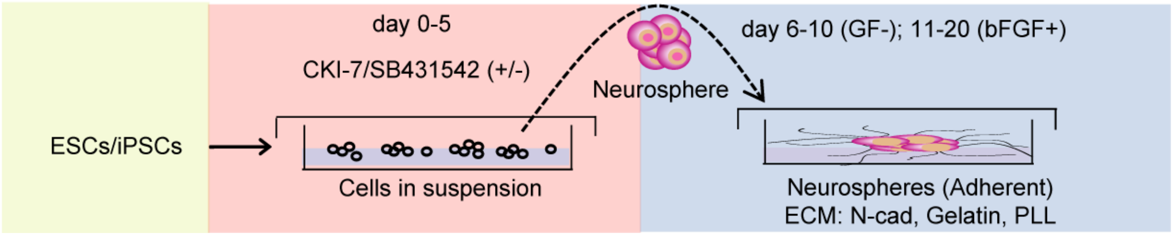

Figure S1. Schematic representation of the neuronal differentiation. ESCs/iPSCs were cultured for 5 days in suspension with and without neuro-inductive soluble factors (CKI-7 and SB-431542). Neurospheres were transferred at day 6 in tissue culture plates pre-coated with extracellular matrix (ECM) proteins and cultured for additional two weeks. Abbreviation: N-cad, N-cad-Fc; PLL, poly-L-lysine; GF, growth factor; bFGF, basic fibroblast growth factor.
