# Supplementary material for: An Engineered N-Cadherin Substrate for Differentiation, Survival, and Selection of Pluripotent Stem Cell-Derived Neural Progenitors
Source: PLoS One. 2015 Aug 5;10(8):e0135170. doi: 10.1371/journal.pone.0135170 (PMC4526632; doi:10.1371/journal.pone.0135170)
Supplement: S2 Fig — (PDF) [file pone.0135170.s002.pdf]

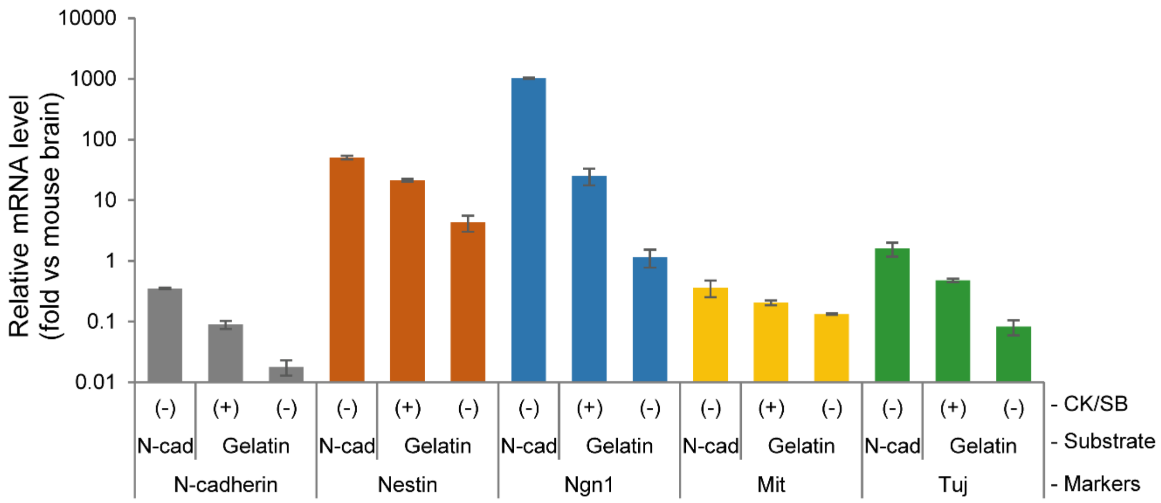

Figure S2. Characterization of ESC-derived neuronal cells differentiated for 20 days on N-cadherin- and gelatin-coated substrate. The relative mRNA expression level of neuronal progenitor cell markers, including N-cad, Nestin, Ngn1, Mitf and Tuj was analyzed by qPCR. Abbreviation: N-cad: N-cad-Fc; Ngn1: Neurogenin 1; Mitf: Microphthalmia-associated transcription factor.
