# Supplementary material for: An Engineered N-Cadherin Substrate for Differentiation, Survival, and Selection of Pluripotent Stem Cell-Derived Neural Progenitors
Source: PLoS One. 2015 Aug 5;10(8):e0135170. doi: 10.1371/journal.pone.0135170 (PMC4526632; doi:10.1371/journal.pone.0135170)
Supplement: S3 Fig — (PDF) [file pone.0135170.s003.pdf]

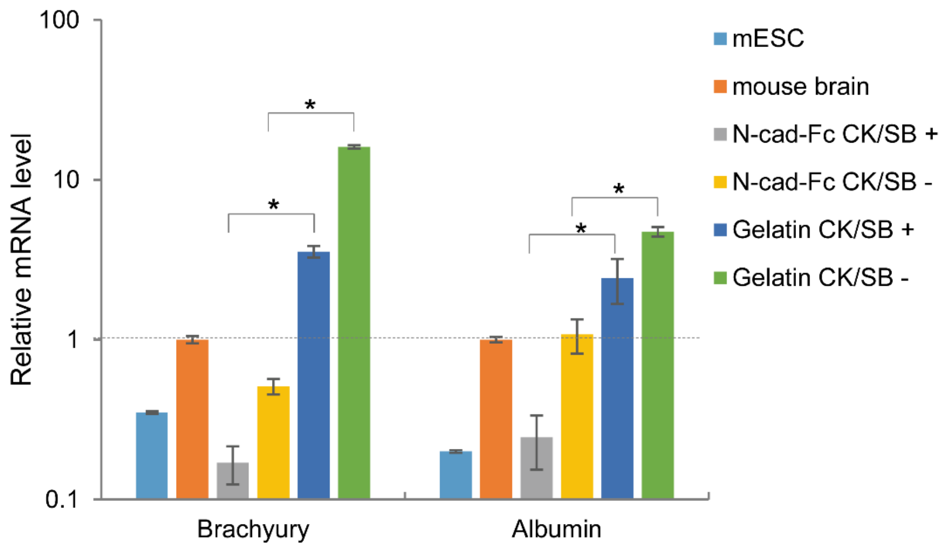

Figure S3. Endoderm and mesoderm marker expression signature of differentiated ESCs. In comparison to mouse brain, the expression of markers for mesoderm (Brachyury) and endoderm (Albumin) progenitors was almost null in differentiated cells cultured with and without CK/SB on N-cadherin substrate. ESCs were also used as control. *: $p<0.05$.
