# Supplementary material for: An Engineered N-Cadherin Substrate for Differentiation, Survival, and Selection of Pluripotent Stem Cell-Derived Neural Progenitors
Source: PLoS One. 2015 Aug 5;10(8):e0135170. doi: 10.1371/journal.pone.0135170 (PMC4526632; doi:10.1371/journal.pone.0135170)
Supplement: S4 Fig — (PDF) [file pone.0135170.s004.pdf]

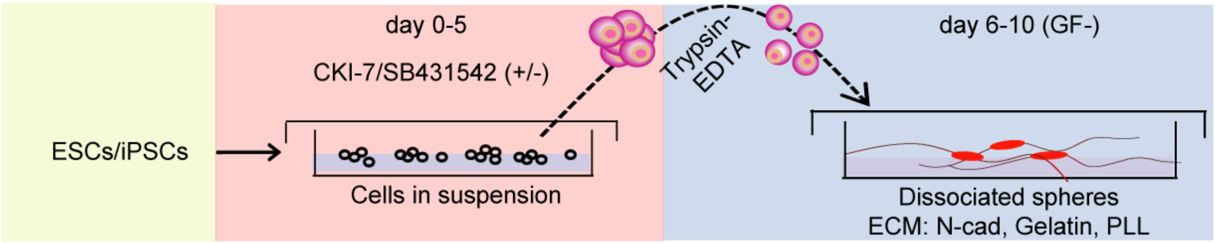

Figure S4. Schematic representation of the modified neuronal differentiation procedure from mouse ESCs/iPSCs in order to enhance the homogeneity of differentiation. ESCs/iPSCs were cultured for 5 days in suspension culture with and without CK/SB. At day 6 the neurospheres were dissociated with trypsin-EDTA and cultured on N-cad-Fc, PLL, and gelatin for 48 hours. Abbreviation: PLL, poly-L-lysine.
