# Supplementary material for: An Engineered N-Cadherin Substrate for Differentiation, Survival, and Selection of Pluripotent Stem Cell-Derived Neural Progenitors
Source: PLoS One. 2015 Aug 5;10(8):e0135170. doi: 10.1371/journal.pone.0135170 (PMC4526632; doi:10.1371/journal.pone.0135170)
Supplement: S5 Fig — (PDF) [file pone.0135170.s005.pdf]

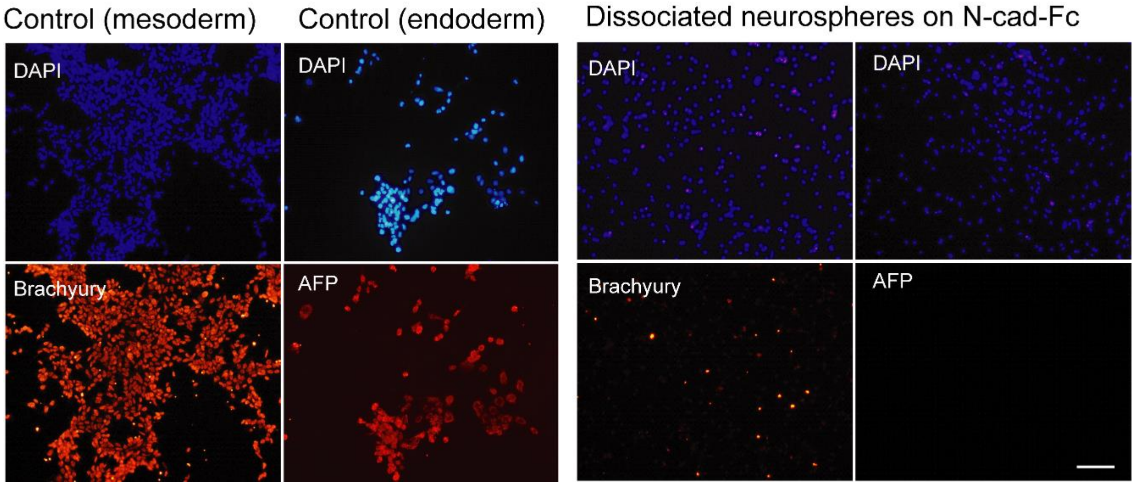

Figure S5. Endoderm and mesoderm markers in differentiated cells upon dissociation. Day 5 neurospheres were dissociated and plated on N-cadherin for 2 days. The cells were immunostained for mesoderm (Brachyury) and hepatic endoderm (AFP) markers. The differentiation-inducing medium used for control experiments were as described [1,2]. Scale bar: 50 μm. α-fetoprotein: AFP.

References

1. Haque A, Hexig B, Meng Q, Hossain S, Nagaoka M, Akaike T (2011) The effect of recombinant E-cadherin substratum on the differentiation of endoderm-derived hepatocyte-like cells from embryonic stem cells Biomaterials 32: 2032-42.
2. Minato A, Ise H, Goto M, Akaike T (2012) Cardiac differentiation of embryonic stem cells by substrate immobilization of insulin-like growth factor binding protein 4 with elastin-like polypeptides Biomaterials 33: 515-23.
