# Supplementary material for: An Engineered N-Cadherin Substrate for Differentiation, Survival, and Selection of Pluripotent Stem Cell-Derived Neural Progenitors
Source: PLoS One. 2015 Aug 5;10(8):e0135170. doi: 10.1371/journal.pone.0135170 (PMC4526632; doi:10.1371/journal.pone.0135170)
Supplement: S6 Fig — (PDF) [file pone.0135170.s006.pdf]

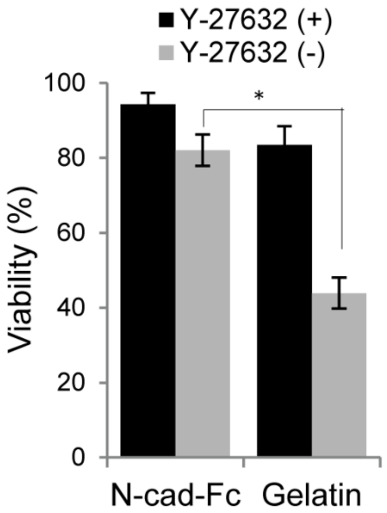

Figure S6. N-cadherin inhibited dissociation-induced-apoptosis in iPSCs. Neurospheres were dissociated on 5 days of differentiation in suspension and plated on N-cad-Fc and gelatin in the presence and absence of ROCK-inhibitor, Y-27632. Cell viability was assessed 24 h after plating. * : $p<0.05$.
