# Supplementary material for: An Engineered N-Cadherin Substrate for Differentiation, Survival, and Selection of Pluripotent Stem Cell-Derived Neural Progenitors
Source: PLoS One. 2015 Aug 5;10(8):e0135170. doi: 10.1371/journal.pone.0135170 (PMC4526632; doi:10.1371/journal.pone.0135170)
Supplement: S1 Table — (PDF) [file pone.0135170.s007.pdf]

Table S1. Primer sequences used in qPCR

Gene	Sequence (forward/reverse)
Nanog	GAGGAAGCATCGAATTCTGG<br>AAGTTATGGAGCGGAGCAGC
N-cadherin	CAGTCTTACCGAAGGATGTGC<br>ATCAGCTCTCGATCCAGAGG
Nestin	GCTACATACAGGACTCTGCTG<br>AAACTCTAGACTCACTGGATTCT
Neurogenin1 (Ngn1)	CGATCCCCTTTTCTCCTTTC<br>TGCAGCAACCTAACAAGTGG
βIII-tubulin	AGCGATGAGCACGGCATAG<br>CAGGTTCCAAGTCCACCAGA
GFAP	GGAGAGGGACAACTTTGCAC<br>GCTCTAGGGACTCGTTCGTG
Mitf	AGAGTCTGAAGCAAGAGCACTG<br>TGCGGTCATTTATGTTAAATCTTC
Nurr	TGAAGAGAGCGGAGAAGGAGATC<br>TCTGGAGTTAAGAAATCGGAGCTG
CHAT	GTAACAGCCCAGGAGAGCAG<br>GCAGGGCTAGAGTTGACTGG
SLC17A6	GGTTCGATGACGTTTCTGGT<br>TCTCGGTTGTCCTGCTTCTT
GAD2	GGGATGTCAACTACGCGTTT<br>TACAGGGGCGATCTCATAGG
Brachyury	CCATGCTGCAGTCCCATGA<br>GCTCACAGACCAGAGACTGGGATAC
Albumin	AGGCTACAGCGGAGCAACTGA<br>TTTGCATCTAGTGACAAGGTTTGGA
β-actin	CCTAAGGCCAACCGTGAAAAG<br>TCTTCATGGTGCTAGGAGCCA
